# Preparation and cell imaging of a *nido*-carborane fluorescent complex based on multi-component polymerization

**DOI:** 10.1371/journal.pone.0313661

**Published:** 2024-12-12

**Authors:** Hezhong Ouyang, Zhou Wang, Min Liu

**Affiliations:** 1 The People’s Hospital of Danyang, Affiliated Danyang Hospital of Nantong University, Zhenjiang, P.R. China; 2 School of Vanadium and Titanium, Panzhihua University, Panzhihua, P.R. China; Maulana Abul Kalam Azad University of Technology West Bengal, INDIA

## Abstract

The biocompatibility of carborane was a difficult problem that had drawn a lot of study interest. Using multi-ion inlay binding, water-soluble polymers were created by encapsulating *nido*-carborane in diazaspirodecaniums such as para-poly-*nido*-carboanylazaspirodecanium [5,4] (p-PNC54), para-poly-*nido*-carboanylazaspirodecanium [6,5] (p-PNC65), meta-poly-*nido*-carboanylazaspirodecanium [5,4] (m-PNC54), and meta-poly-*nido*-carboanylazaspirodecanium [6,5] (m-PNC65). First, the active control 5-fluorouracil demonstrated strong activity against HeLa and HCT-116 cells but minimal cytotoxicity at 19.22±2.85 μM and 21.47±5.99 μM, respectively. Second, the four carborane polymers that specifically penetrated the cells were imaged using HeLa cells. TEM was used to assess the dynamic self-assembling effect of these water-soluble polymers in order to gain a better understanding of their internal microphenomenon. All four derivatives’ combined impacts on self-assembly in water were identified. Different degrees of selective entrance into targeted cells under different architectures were discovered by in vitro cell imaging.

## 1. Introduction

In 1936, Professor Gordon Locher first proposed the idea of boron neutrons, soon afterwards, the concept of Boron Neutron Capture Therapy (BNCT) came into being [1–3]. As part of this cutting-edge tumor radiation therapy, a safe targeted material (boron) was injected into the body to create a selective nuclear microreactor in targeted matter, which effectively killed cancer cells without endangering healthy ones [4,5].

**Fig 1:**
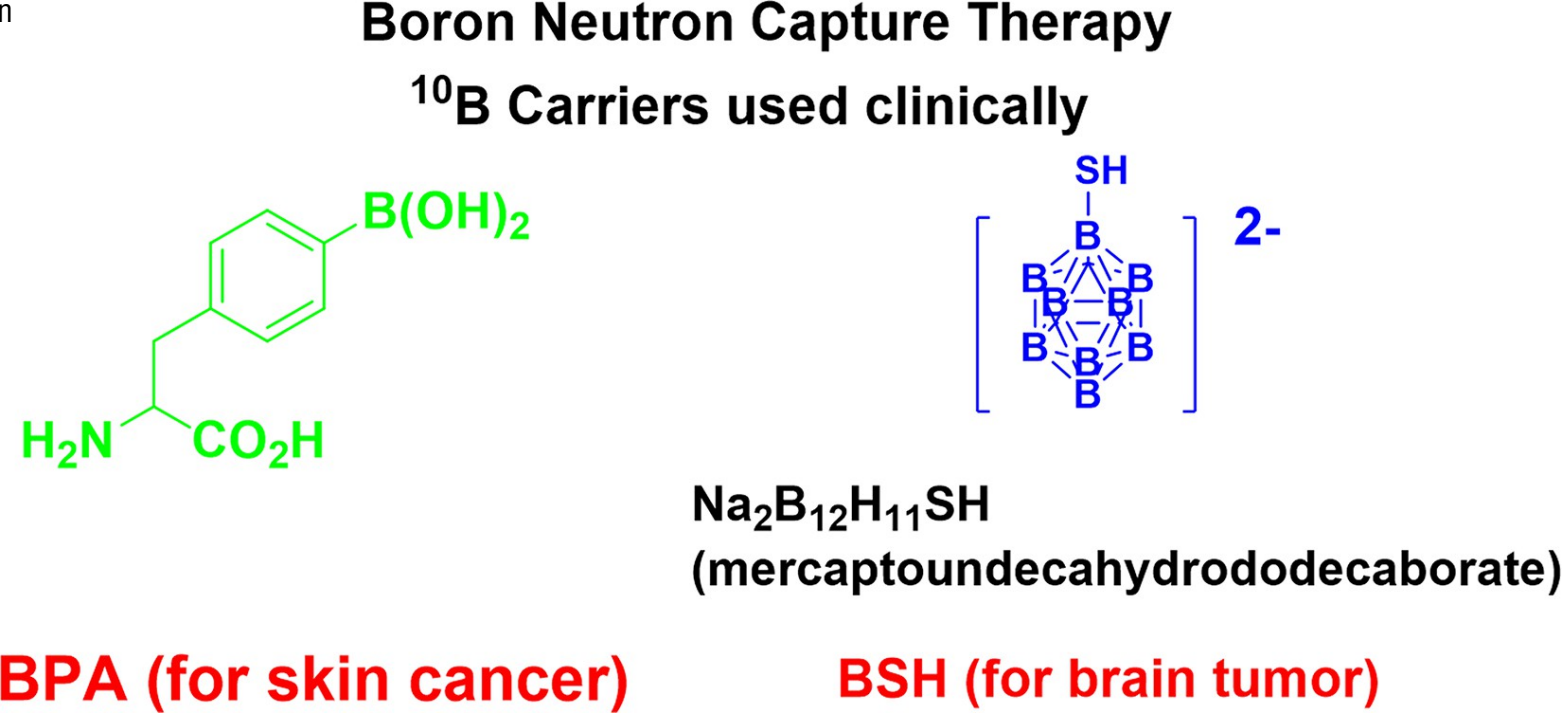
Chemical structures of BPA and BSH.

Currently, boron-containing compounds used in BNCT must either accumulate preferentially in cancer tissue or be non-toxic or have very low toxicity when administered at therapeutic levels. Although Sodium borocaptate (BSH) and boronophenylalanine (BPA) (Fig 1) have low levels of chemical toxicity, they do not satisfy the boron concentration requirements [6–8]. It is necessary to develop a new boron-containing compound that can improve drug concentration in both diseased and healthy cells [9–12], and satisfy the present treatment requirements of BNCT in order to address the aforementioned shortcomings of BPA and BSH [13–15]. Environmental elements linked to water pollution and a variety of medications high in organoborane also need to be introduced [16–24].

Nevertheless, because of their strong affinity for tumor cells, carboranes can specifically enter them [25–27]. The low biocompatibility and low solubility of most carboranes limit their applicability in BNCT even further. Despite notable advancements in water solubility in BNCT experiments, the blood-brain barrier (BBB) still poses challenges [28–30]. Moreover, carboranes’ distribution and the proportion of blood drug concentration that is targeted toward the cells can be approximated, even though it is impossible to see the precise amount of carboranes entering the cells. Therefore, BNCT research urgently needs a highly selective targeted cell fluorescence probe to estimate blood drug concentration and rapidly determine their distribution [31–35].

The search for potent carboranes to effectively address the issues that currently accompany organoboranes led to the development of several low-toxicity and high-activity dynamic self-assemblies for targeted fluorescence placement. By using fluorescence to specifically target cells, this strategy aims to enhance drug-induced self-assembly and achieve infiltration. It significantly improves carboranes’ water solubility in vivo, enhancing biocompatibility, while also resolving the drug’s toxicity issue. Furthermore, it promotes specific drug aggregation within cells to achieve effective therapeutic outcomes.

## 2 Experimental

### 2.1 General materials

All solvents and reagents were purchased commercially and used without further purification. The used reagents are carborane (96%, Katchem), potassium hydroxide (98%, RG), and acid solvent (RG), which were purchased through commercial channels such as Titan Technologies. The reaction process was monitored by thin layer chromatography (TLC), and the TLC results were analyzed under UV lamps at 254 and 365 nm. On the Bruker Avance II spectrum analyzer of MeOD (400 MHz is ^1^H), the characteristics of each product were recorded by ^1^H NMR spectroscopy: the chemical shift was expressed in ppm, and tetramethylsilane (TMS) was used as the internal standard. The morphologies were observed with a transmission electron microscopy (TEM). The TEM was measured using a field emission transmission electron microscopy JSM-7001F (JEOL, Japan).

### 2.2 Chemistry

The general procedure for creating m-PNC54, m-PNC65, p-PNC54, and p-PNC65 polymers was as follows. As an example, 10 mL of EtOH were used to dissolve 2-acetoxybenzoic acid (2.1 eq) and NaOH (2.4 eq). The mixture was then stirred for eight hours at room temperature while terephthalaldehyde (1.0 eq) was added. The process of reaction was observed through TLC. After the end of the reaction, the mixture was filtered, and the solid product was cleaned with three parts EtOH, 3 x mL, and then dried. Compound 1a was the result, a pale orange solid with a yield of 60% and a weight of 0.49 g.

0.2 g para-Carboxybetaine methacrylate (p-CBMA) was dissolved in 5 mL of MeOH, and 1a (0.2 g, 0.4 mmol) was added and reacted at 40°C for 48 hours. The white solid precipitated, and then the reaction mixture was filtered to obtain the milky white oily solid para-poly-carboxylazaspirodecanium (p-PCC), weighing 0.37 g without further purification. The polymer p-PCC (0.2 g) and compound 2a (0.1 g) were dissolved in 5 mL of MeOH at 40°C for 72 hours, followed by concentration of the reaction solution to obtain a gray-white oily solid p-PNC54.

### 2.3 Typical procedure for measuring the UV–vis spectra

Using a quartz cuvette with a 1 cm route length and room temperature, a UV-2550 spectrophotometer was used to record ultraviolet-visible (UV-Vis) spectra. The samples were produced as 5 mM stock solutions in DMSO, MeOH, EtOH, and H_2_O. These solutions were then diluted to achieve the desired concentration. Within a scan range of 200 to 400 nm, the UV-Vis absorption spectra were obtained with H_2_O, MeOH, EtOH, and DMSO acting as controls.

### 2.4 Typical procedure for collecting fluorescence spectra

Fluorescence spectra were performed using a Shimadzu RF-5301PCS spectrofluorophotometer. A stock solution of samples (5 mM) was prepared in H_2_O, MeOH, EtOH, and DMSO, and subsequently diluted to obtain the appropriate concentration. Fluorescence spectra were recorded at wavelengths ranging from 200 to 600 nm with an excitation wavelength of 400 nm.

### 2.5 MTT assay

HeLa, HCT-116, and normal liver L-02 cells were screened for cytotoxicity in vitro. All the cells were obtained from the American Type Culture Collection (USA). HeLa and HCT-116 cells were routinely cultured in RPMI-1640, while L-02 cells were routinely cultured in DMEM. The media were supplemented with 10% fetal bovine serum (FBS), and the cells were incubated in a humidified atmosphere at 37°C with 5% CO_2_. These cells were monitored daily and maintained at 80 percent cell density.

The cytotoxicity of each cell line was measured during the logarithmic growth stages in MTT cancer cells (HeLa and HCT-116) and normal lung L-02. All cells were inoculated into a 96-well plate at a rate of 106 cells per well. The samples were then treated with different concentrations of 5-F (1, 3, 5, or 10 mmol/mL) and tested for 24 hours. The supernatant was dissolved in 100 mL DMSO and shaken well for 10 minutes. The optical density of the sample was measured at 490 nm using a microplate photometer. Cell activity was expressed as a percentage change in absorbance relative to the control value.

### 2.6 Living cell imaging

HeLa cells in the logarithmic growth phase were trypsinized, inoculated into 6-well plates containing round coverslips, placed in an incubator at 5% CO_2_, and cultured at 37°C for 24 h until adhering to the wall. A stock solution of carborane polymers (20 mg/mL) was prepared in DMSO and subsequently diluted with DMSO to obtain the appropriate concentration. The original culture solution of cells in each well was removed and replaced with a medium containing different samples at a concentration of 10 μg/ml. The cells were then treated for 24 h. After that, the medium was discarded, and the wells were washed twice with PBS. Paraformaldehyde fixing solution was added to fix the cells for 10 min. Following removal of the fixing solution and two additional washes with PBS, the cells were incubated with DAPI in darkness for 10 min. Finally, the staining solution was discarded, and another two washes with PBS were performed before obtaining cell fluorescence images using a fluorescence microscope after treating them with anti-fluorescence quenched mounts.

### 2.7 Characterization

Transmission Electron Microscope (TEM) was performed on a Zeiss Ultra Plus at an accelerating voltage of 15 keV, with an attached Oxford Instruments X-Max 60 mm^2^ SDD X-ray microanalysis system. The ethanol-suspended precipitate of the sample was added to a silicon wafer, and the sample was attached to a sample tray with conductive adhesive. TEM images were obtained using a scanning electron microscope with rulers measuring 0.5 μm and 200 nm, respectively.

## 3. Result and discussion

### 3.1 Design and synthesis

**Scheme 1:**
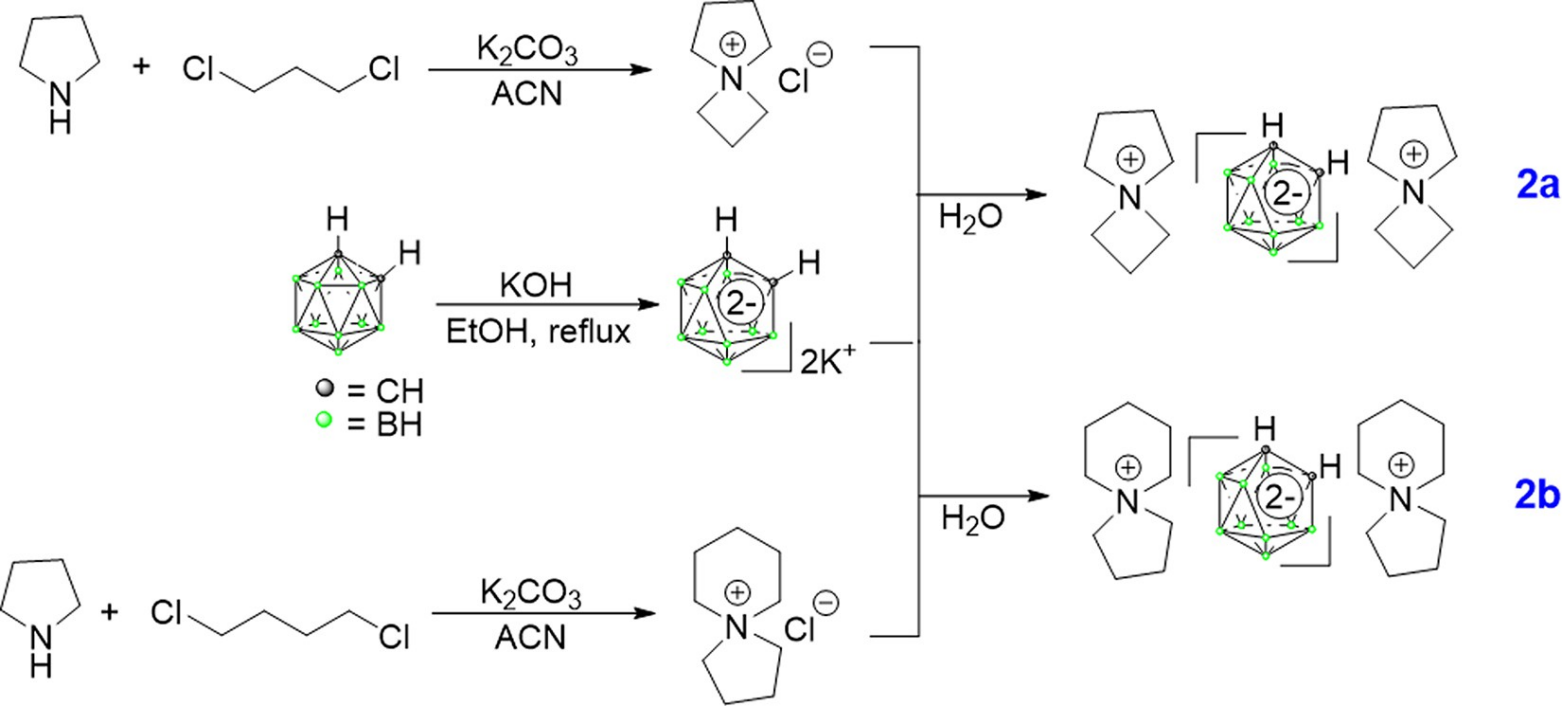
Chemical structures of azaspirodecanium [4,5] and azaspirodecanium [5,6].

**Scheme 2:**
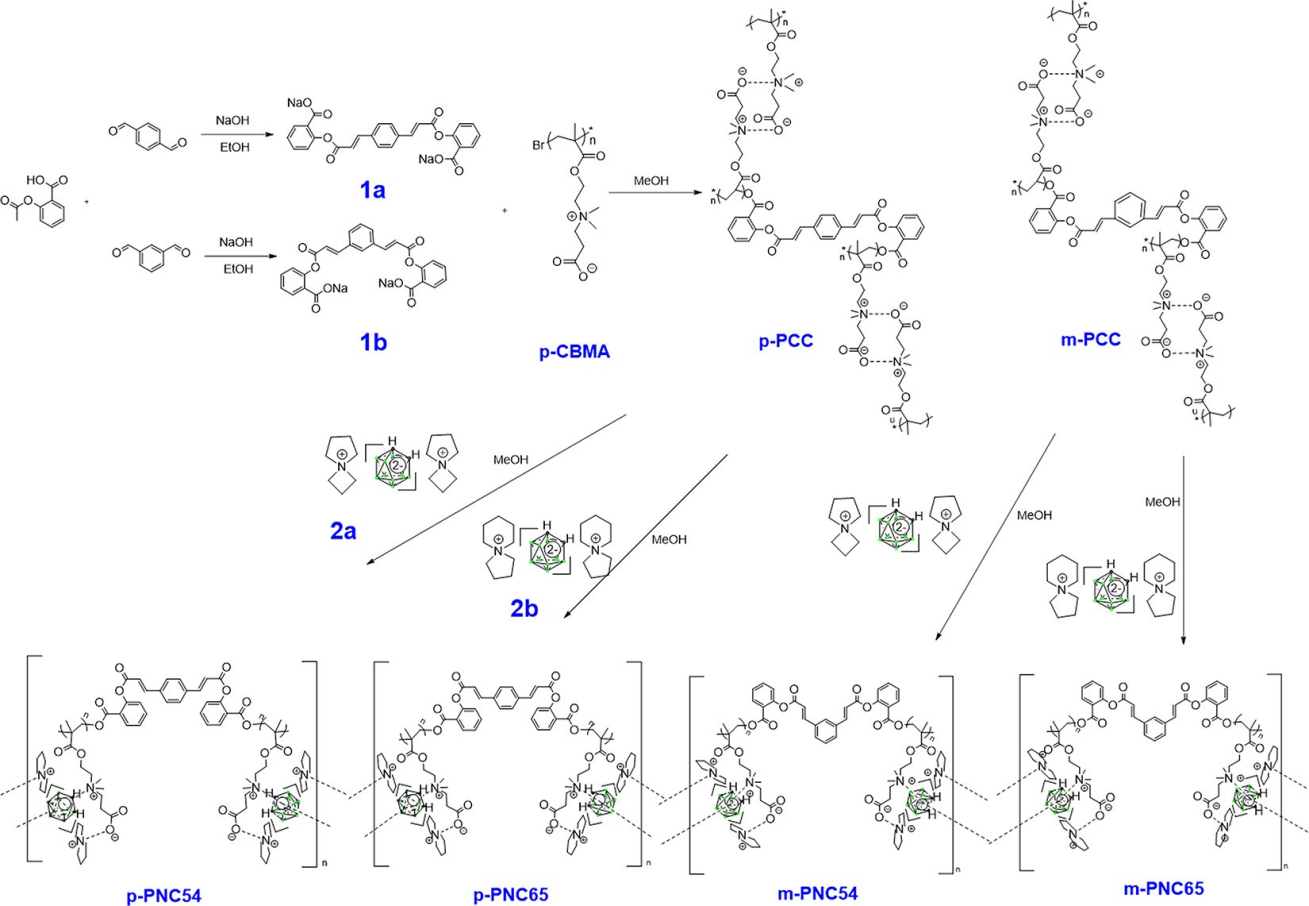
Chemical structures of p-PNC54, p-PNC65, m-PNC54, and m-PNC65.

The synthetic layout was logically designed with consideration for four advances. The materials known as azaspirodecanium [4–6] were synthesized and were widely utilized in lithium battery electrolytes due to their high solubility. 2) A common double ion in transition metal-ligand chemistry (Scheme 1) was nido-carborane [36]. Because of its high water solubility, it was produced by refluxing *o*-carborane in a strong alkali. 3) When creating a polymer carrier, the water-soluble intermediate p-CBMA [37] was widely used to treat the brain’s blood-brain barrier, significantly boosting the material’s overall biocompatibility. Utilizing the condensation process, p-PCC and m-PCC were prepared. In the end, azaspirodecanium [4,5] and azaspirodecanium [5,6] were added to carborane polymers (p-PCC and m-PCC) using their multi-ionic bonding properties; their hydrogen spectra were shown in S1 and S2 Figs. Four extremely soluble in water *o*-carborane derivatives were the end product of Scheme 2.

### 3.2 Photophysical properties

**Fig 2:**
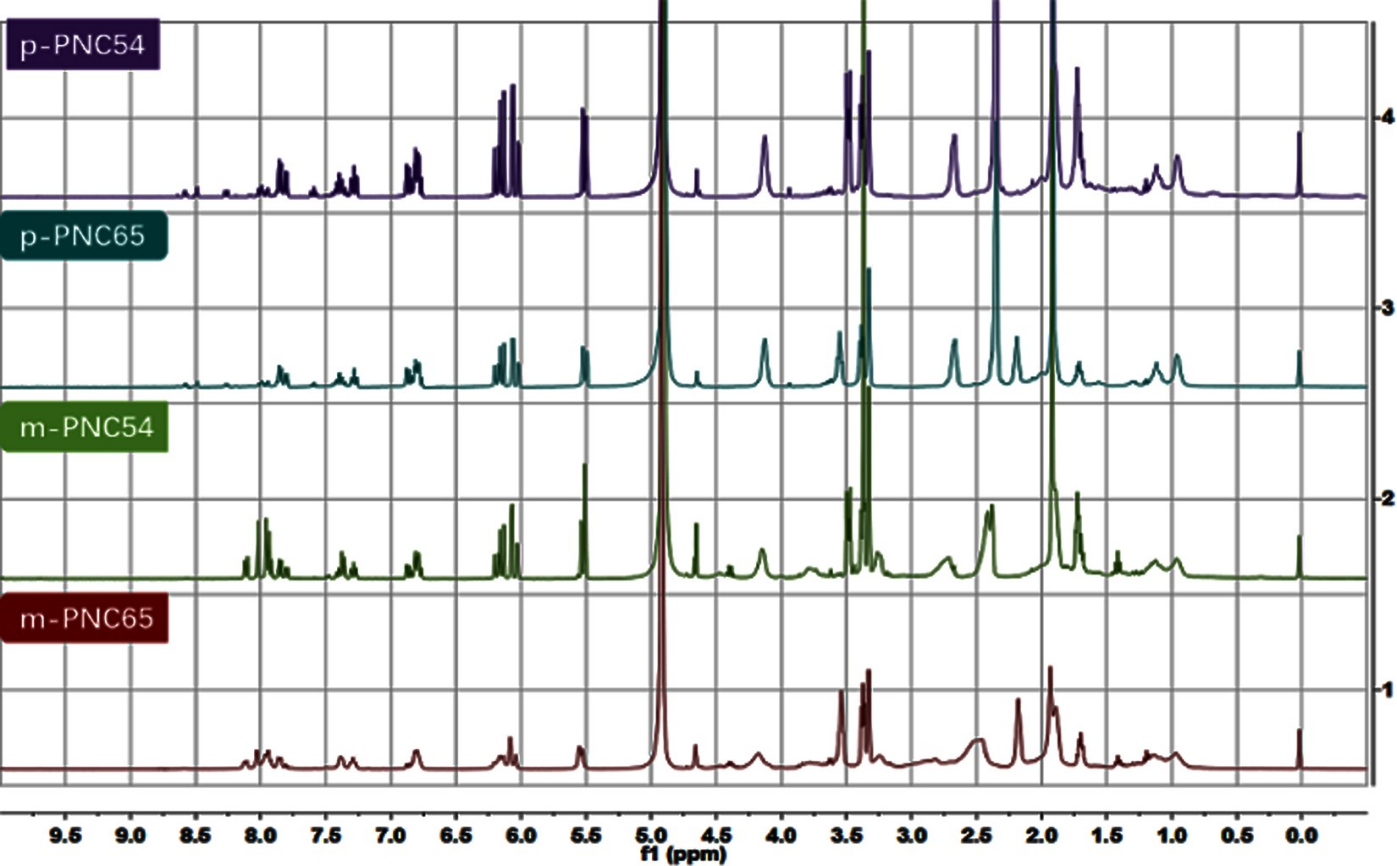
^1^H-NMR spectra of p-PNC54, p-PNC65, m-PNC54, and m-PNC65.

A preliminary structural characterization of the four carborane polymers was determined by using nuclear magnetic resonance (NMR) spectroscopy to analyze the characteristic peaks of the two main functional groups. Figure’s hydrogen spectra (Fig 2) showed several aromatic characteristic peaks between 6.0 and 8.5 ppm. (The matching single hydrogen spectra were shown in S3 through S6 Figs). Convex peaks were clearly visible in the high magnetic field region between 0 and 3 ppm, and because of their superposition with the polymer branch chain peaks typical BSH groups irregular cluster peaks were also discernible.

**Fig 3:**
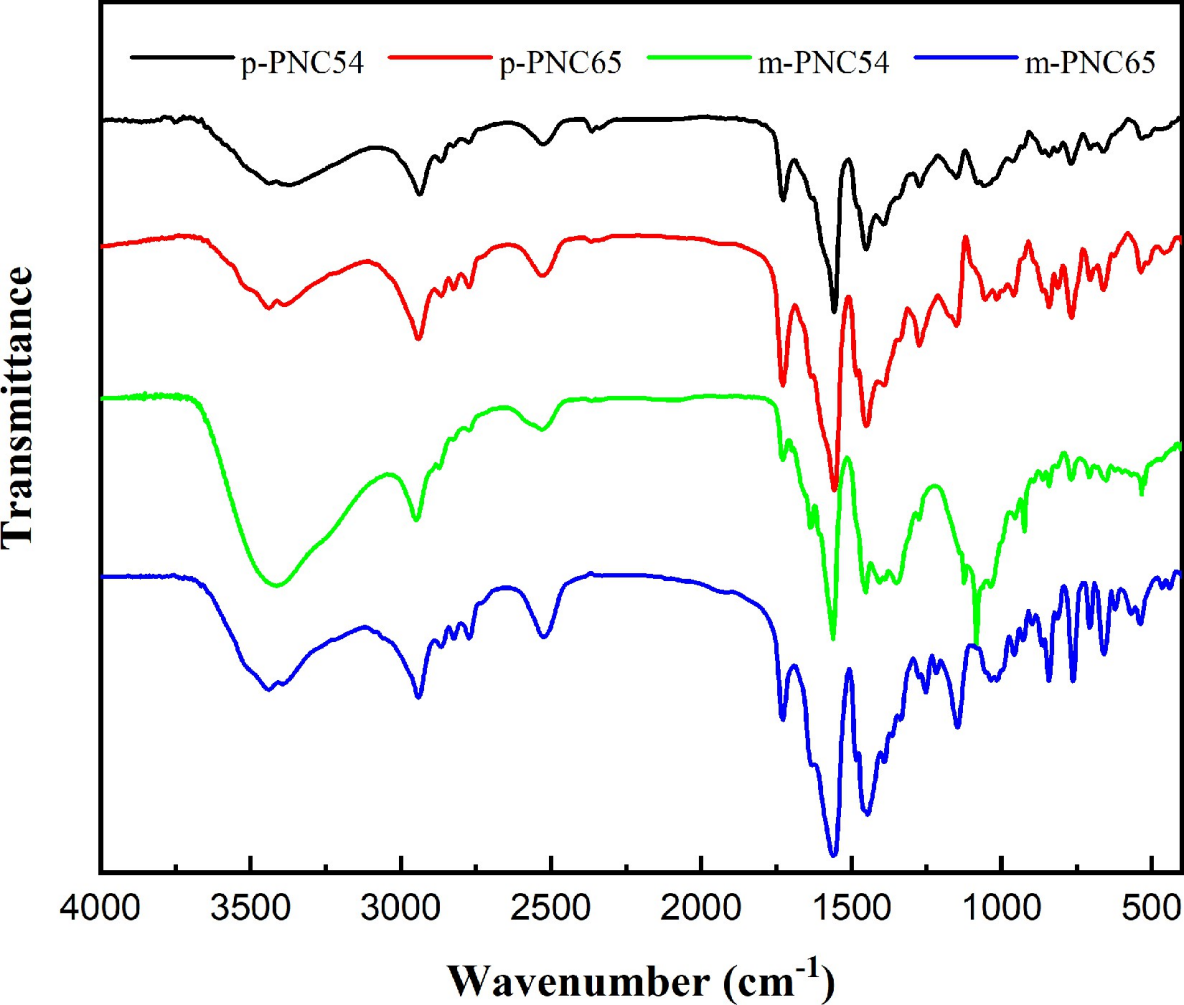
FTIR spectra of p-PNC54, p-PNC65, m-PNC54, and m-PNC65.

Two functional group peaks are visible after a thorough analysis of four carborane polymers using infrared spectroscopy. A strong peak of absorption was found at about 1500 cm^-1^. As the distinctive peak of several aromatic groups, this peak was identified (Fig 3, with the matching single FTIR spectra displayed in S7–S10 Figs. The typical carborane absorption peak in the boron spectrum was identified by the smooth protrusions present in the absorption peak at 2530 cm^-1^. A blue shift in the boron absorption peak of the entire carborane was observed, owing to the absence of two boron compounds, although the typical BH peak generally fell between 2560 cm^-1^ and 2600 cm^-1^.

**Fig 4:**
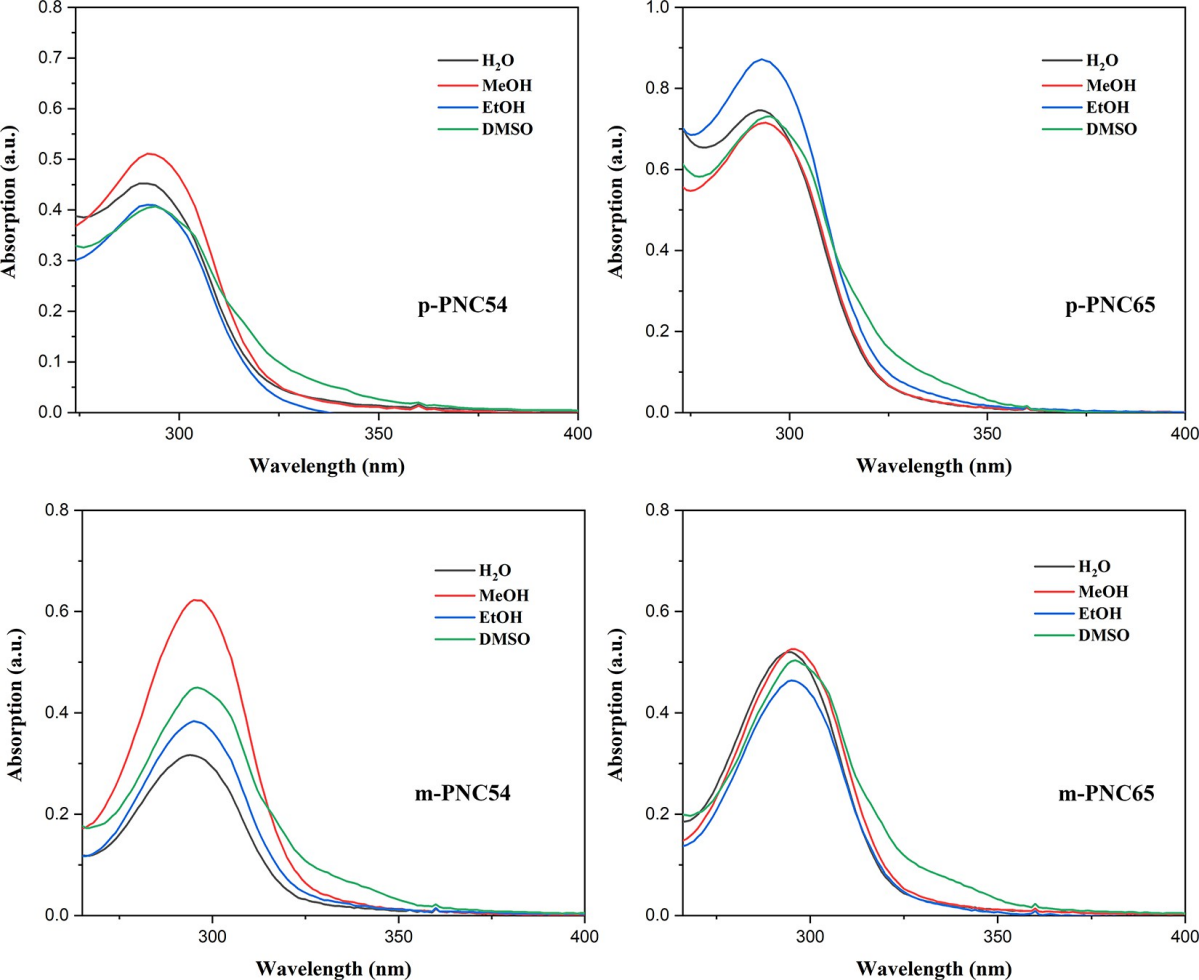
Absorption spectra of p-PNC54, p-PNC65, m-PNC54, and m-PNC65.

Several polar solvents were tested across the spectrum range as spectral media to investigate their photophysical properties in solution more thoroughly. These solvents included water, methanol, ethanol, and DMSO. Each of the four carborane polymers had similar absorption spectra regardless of the solvent used. Most of their spectra fell within the range of 292 nm – 296 nm. As the absorption spectra were primarily concentrated in the structure of the (cinnamoyloxy)benzoate polymer, branching did not significantly affect the functional groups attached to either side of the polymer (Fig 4).

To examine the spectrum range and learn more about the photophysical properties of these polar solvents in solution, a range of spectral media were selected, including water, methanol, ethanol, and DMSO. All four carborane polymers exhibited similar absorption spectra, regardless of the solvents used. It was discovered that the spectra of λabs had a concentration ranging from 292 nm – 296 nm. With little to no impact on the branchate’s functional groups attached to either of the two sides, the absorption spectra’s main focus was the (cinnamoyloxy)benzoate polymer structure overall.

**Fig 5:**
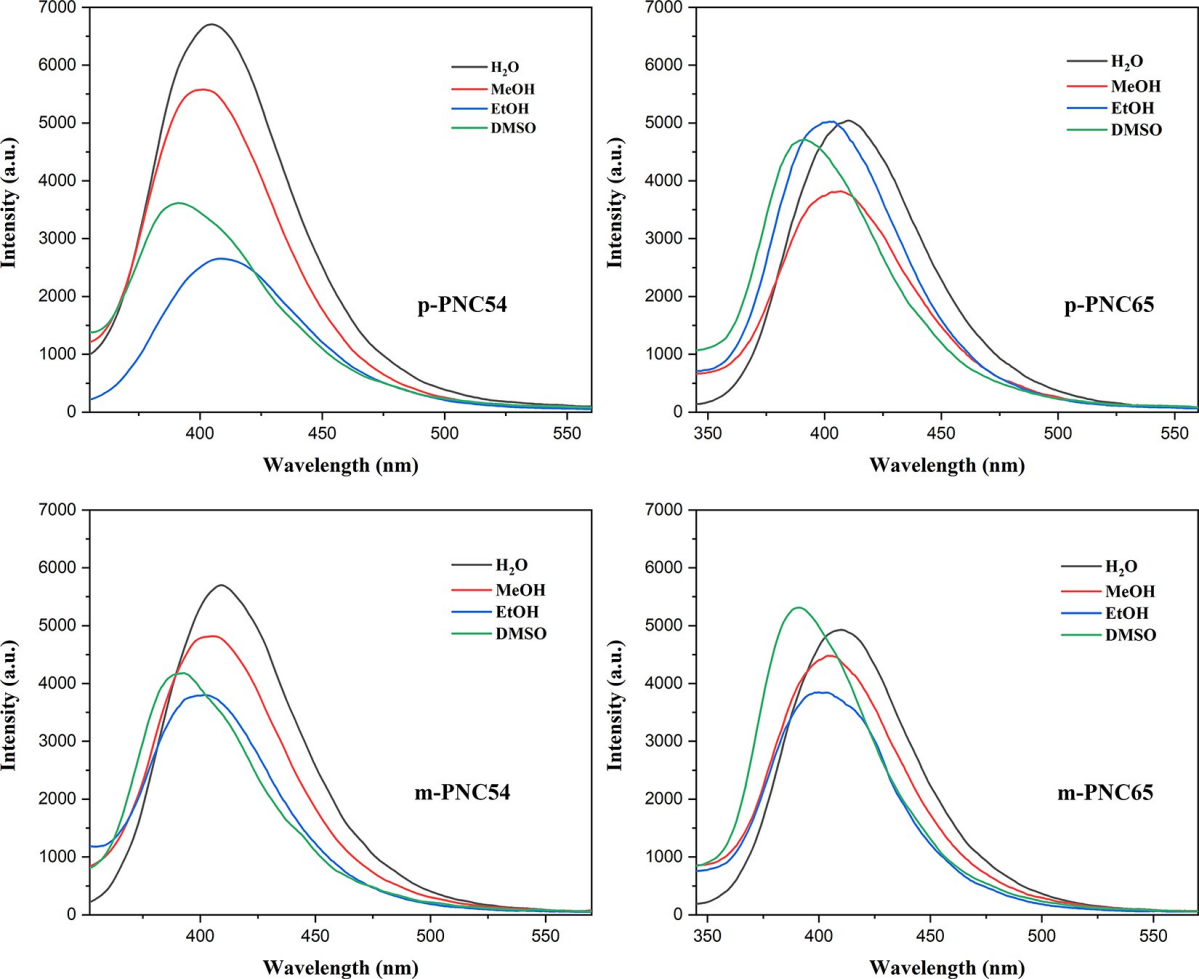
Fluorescence spectra of p-PNC54, p-PNC65, m-PNC54, and m-PNC65.

The fluorescence spectrum and the previous absorption spectra were also different. The emission wavelength of λ_em_ in DMSO exhibits the lowest range, varying between 390 nm and 393 nm. Comparing alcohol solvents to water solvents, there was no appreciable difference in the emission wavelength of λ_em_, which ranged from 400 nm to 409 nm. However, Fig 5 and Table 1 showed a general increase in the wavelength of emission in water.

**Table 1:** Emission wavelengths of p-PNC54, p-PNC65, m-PNC54, and m-PNC65 in different solvents.

Compounds		p-PNC54	p-PNC65	m-PNC54	m-PNC65
λ_abs max_, nm	H_2_O	292	293	294	294
	MeOH	292	294	295	296
	EtOH	292	293	296	295
	DMSO	294	295	296	296
λ_em max_, nm	H_2_O	411	410	407	409
	MeOH	407	403	406	404
	EtOH	409	402	402	404
	DMSO	390	392	393	391

**Fig 6:**
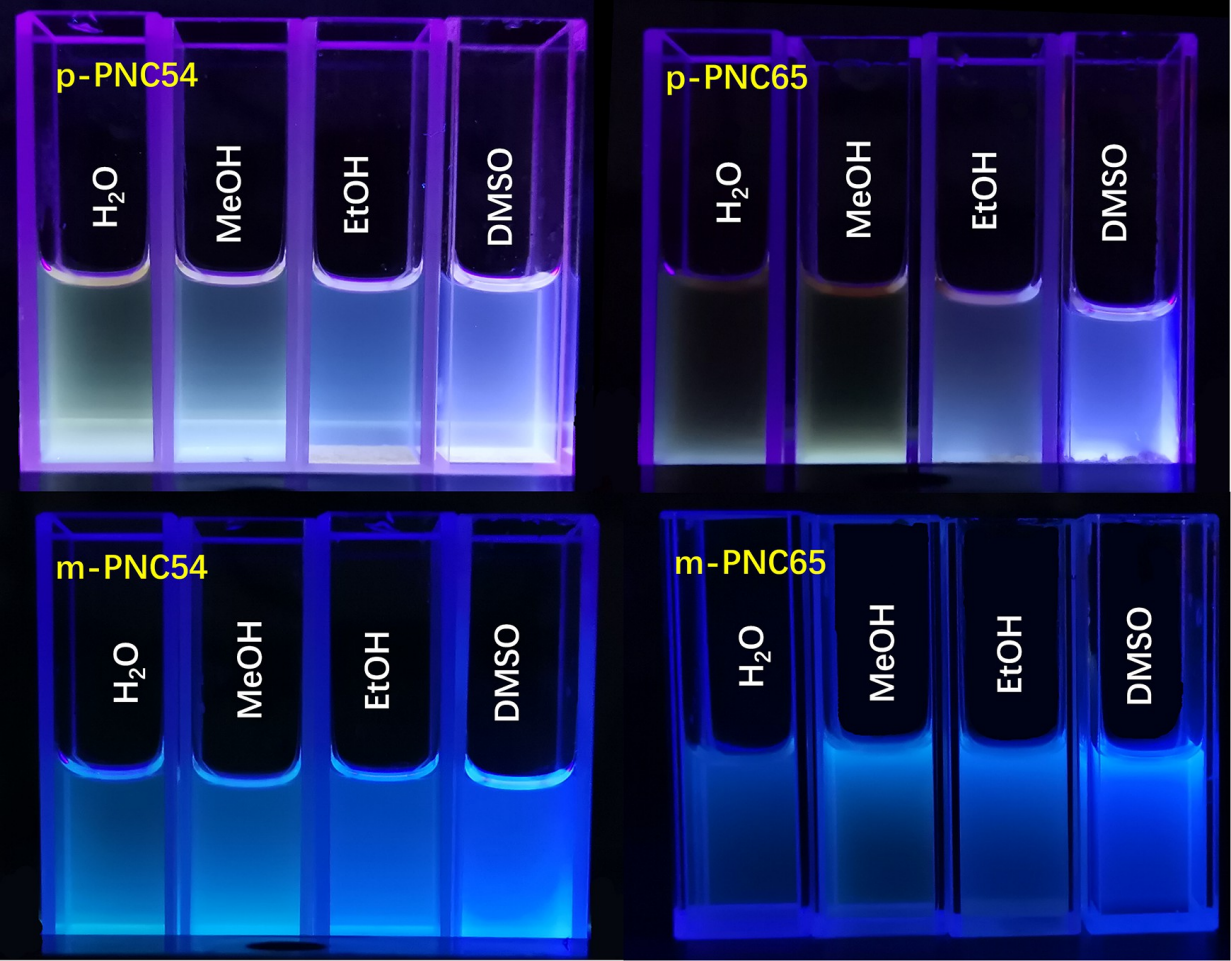
UV images of p-PNC54, p-PNC65, m-PNC54, and m-PNC65 at 365 nm.

Protic and aprotic solvents could affect the polymer structure in the multi-ion inlay form, along with other external solvents. Multiple ion morphology was not significantly affected by the use of the aprotic solvent DMSO in the carborane polymer construction process. In contrast, the fluorescence spectra of water, methanol, and ethanol were similar for protic solvents. The effect was most prominent in water because a protic solvent might provide a proton and form a hydrogen bond with the carbon borane polymer to associate or create a coordination cation (Fig 6).

### Biology

#### 3.3.1 Evaluation of cytotoxicity activity in vitro

**Fig 7:**
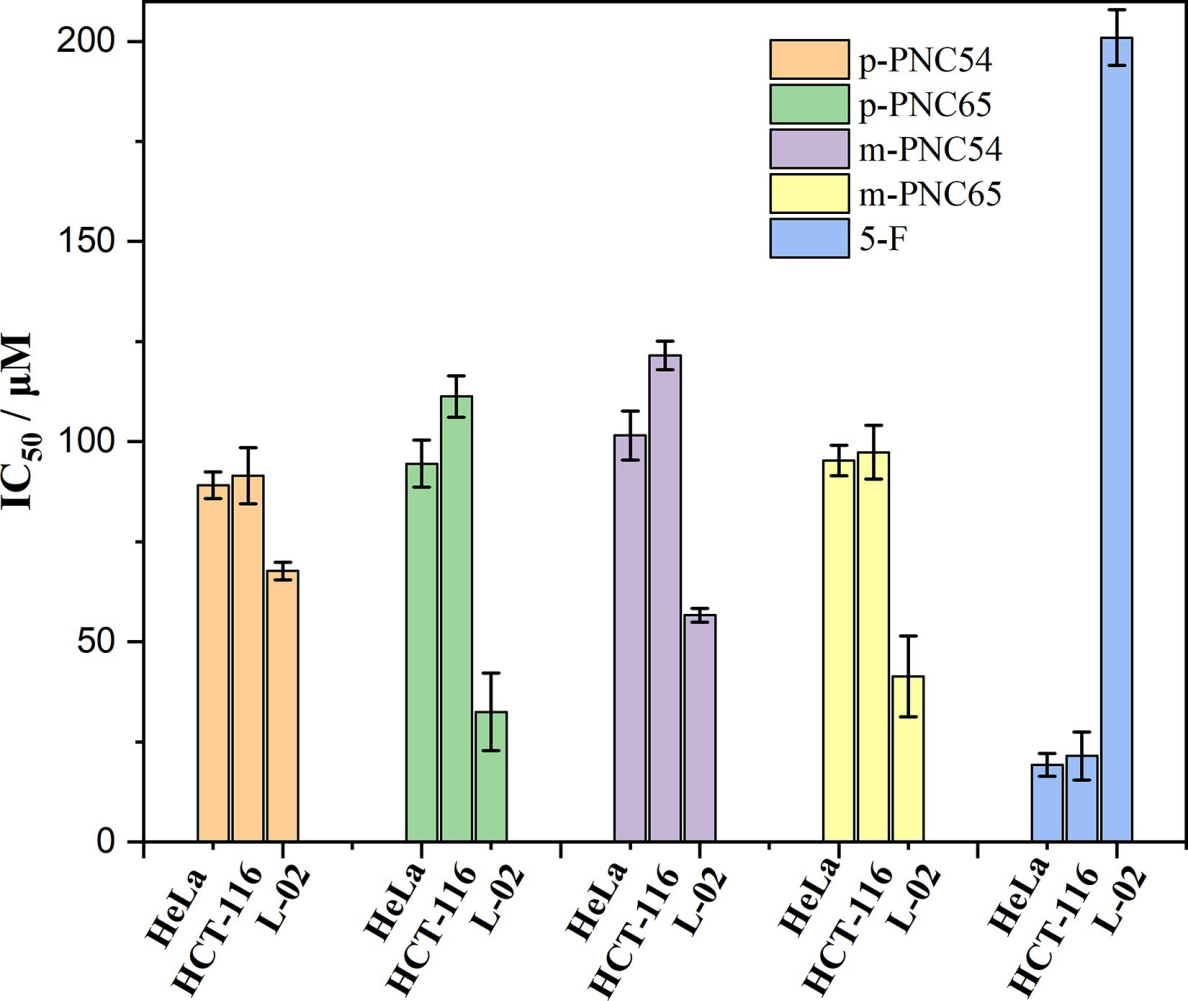
Cytotoxicity activities in vitro of p-PNC54, p-PNC65, m-PNC54, and m-PNC65.

In comparison to 5-fluorouracil as the control, it was discovered that the human cancer cell lines HeLa, HCT-116, and L-02 were the most cytotoxic. Initially, the active control 5-fluorouracil showed excellent activity against HeLa and HCT-116 cells but little cytotoxicity at 19.22±2.85 μM and 21.47±5.99 μM, respectively. On the other hand, although not very reactive overall, the four carborane polymers showed considerable cytotoxicity and moderate activity. Carbanes showed significant toxicity in an in vitro biological analysis; in particular, nido-carborane showed toxicity above its harmful concentration limit. Because of this, water-soluble gel carriers and bis(cinnamoyloxy)benzoate were added during design to reduce their levels of toxicity. As expected, all four derivatives achieved an intermediate level of toxicity in vitro when compared to nido-carborane’s results, but p-PNC54 stood out for its higher levels of activity and cytotoxicity when compared to the other carborane polymers (Fig 7 and Table 2).

**Table 2:** Cytotoxicity activity in vitro of p-PNC54, p-PNC65, m-PNC54, and m-PNC65.

Compounds	IC_50_ (μM) ± SD^a^		
	HeLa	HCT-116	L-02
**p-PNC54**	89.09±3.31	91.44±7.01	67.68±2.25
**p-PNC65**	94.48±5.90	111.29±5.15	32.49±9.67
**m-PNC54**	101.56±6.11	121.52±3.57	56.63±1.77
**m-PNC65**	95.25±3.79	97.36±6.72	41.37±10.11
**5-F**	19.22±2.85	21.47±5.99	>200

a = The deviation is means ± SD, n = 3.

#### 3.3.2 Cell imaging

**Fig 8:**
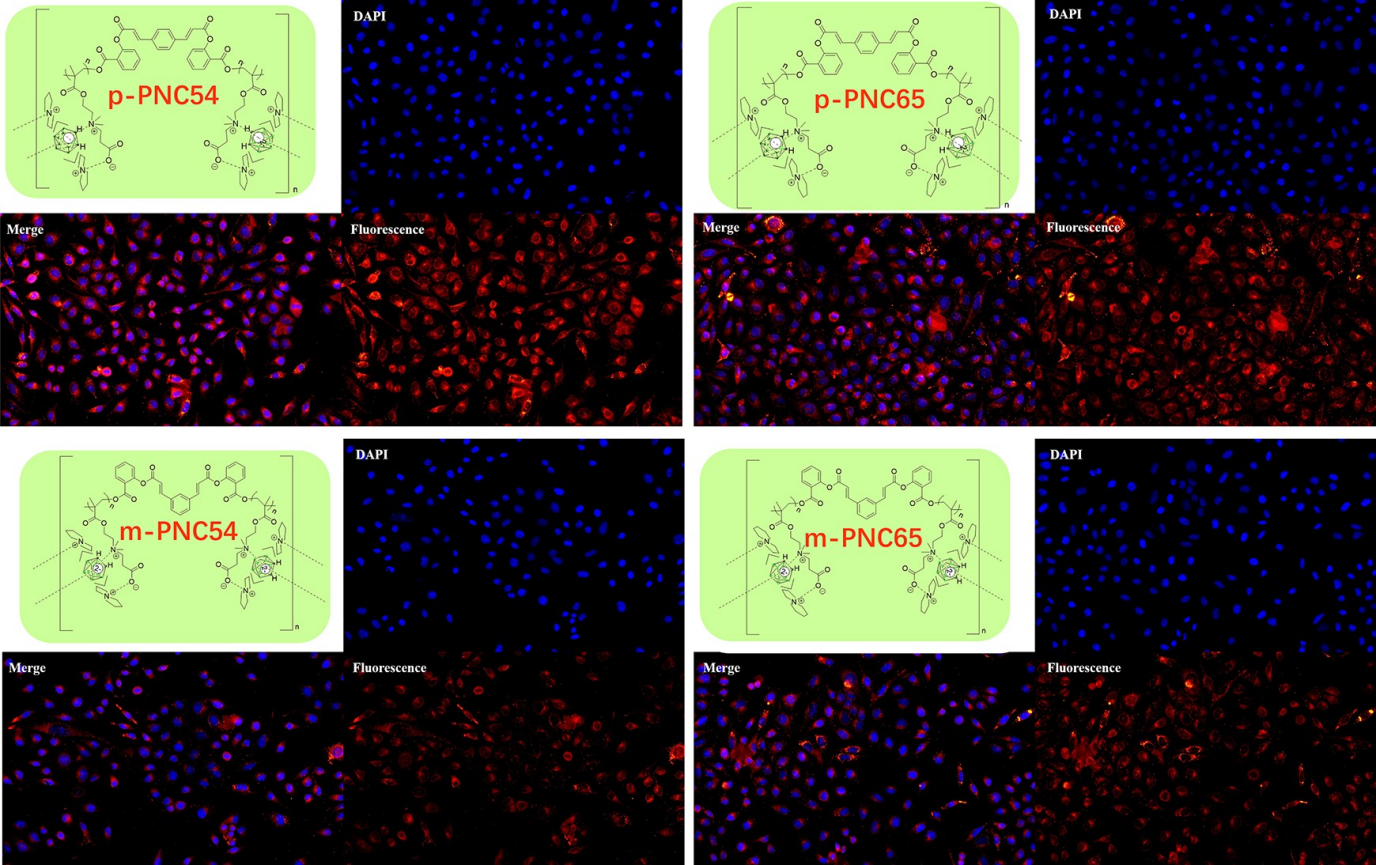
Fluorescence images of p-PNC54, p-PNC65, m-PNC54, and m-PNC65 in HeLa cells.

Cell imaging studies, which were predicated on the structural characterization and toxicity experiments of the four carborane polymers, demonstrated their high selectivity, permeability, and biocompatibility in targeted cell lines. The fluorescence of m-PNC54, m-PNC65, and p-PNC54 and p-PNC65 varied, as shown in Fig 8. It was not implied by this, though, that m-PNC54 and m-PNC65’s selectivity and permeability were inferior to those of p-PNC54 and p-PNC65. Nevertheless, significant differences in fluorescence were found as a result of modifications to electron transport channels produced by aromatic ring arrangements at meta- or para-locations. Though they all entered the cytoplasm of the cells, the majority of the four carborane polymer forms (especially p-PNC54 and p-PNC65) entered the nucleus.

**Fig 9:**
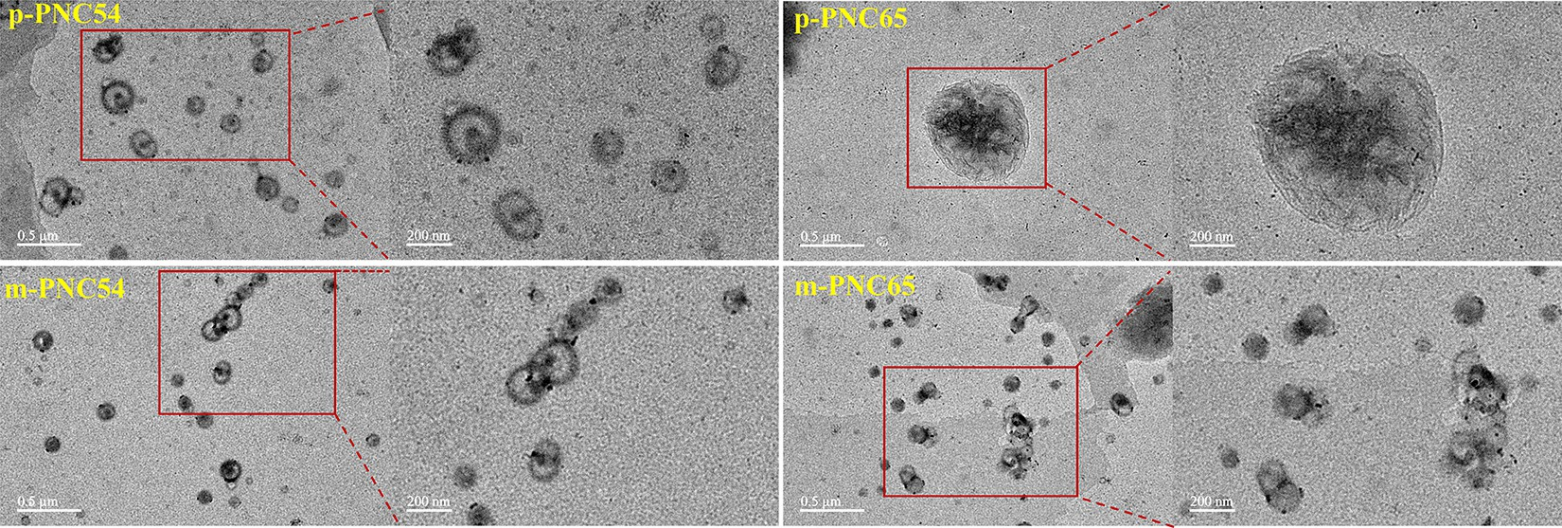
TEM images of p-PNC54, p-PNC65, m-PNC54, and m-PNC65.

The four different types of carborane polymers were examined for their dynamic morphology at the micro-level by using transmission electron microscopy (TEM) to find the corresponding self-assembling trend of each type. The morphology displayed a consistent, solid circular pattern (Fig 9). Both ends of the polymers created multiple ionic bonds that were stable and regular, allowing the molecules to self-assemble, agglomerate, and cross the blood-brain barrier to have targeted cellular effects. In contrast to p-PNC54 and p-PNC65, m-PNC54 and m-PNC65 displayed more pronounced meta-position aggregation, with clusters of nearly infinitely many solid circles together. The difference in the fluorescence effect between the two groups’ aggregation, as seen in Fig 6, may also be explained by this.

## 4 Conclusions

The findings of a thorough analysis of four factors (high permeability, high water solubility, high biocompatibility, and good selectivity of carborane polymers) were reported in this work. Different from the schemes reported in the past, the preparation is simple, the design route is clear, and different series of polyionic polymer drugs can be obtained by one pot method. Moreover, in order to improve the dissolution level and cell selectivity of the water-soluble gel polyionophores, biionic azazadodecane was added in series by multiple ionic bonds. Such design not only solves the application of carborane in the biological field but also greatly increases the concept of nanoborane drug design. This allowed for a variety of applications in BNCT research. These results were important for studying carboranes’ biocompatibility.

## Supporting information

S1 Fig ^1^H-NMR spectrum of p-PCC. (TIF)

S2 Fig ^1^H-NMR spectrum of m-PCC. (TIF)

S3 Fig ^1^H-NMR spectrum of p-PNC54. (TIF)

S4 Fig ^1^H-NMR spectrum of p-PNC65. (TIF)

S5 Fig ^1^H-NMR spectrum of m-PNC54. (TIF)

S6 Fig ^1^H-NMR spectrum of m-PNC65. (TIF)

S7 Fig FTIR spectrum of p-PNC54. (TIF)

S8 Fig FTIR spectrum of p-PNC65. (TIF)

S9 Fig FTIR spectrum of m-PNC54. (TIF)

S10 Fig FTIR spectrum of m-PNC65. (TIF)
