# Supplementary figures and images for: Preparation and cell imaging of a nido-carborane fluorescent complex based on multi-component polymerization
Source: PLoS One. 2024 Dec 12;19(12):e0313661. doi: 10.1371/journal.pone.0313661 (PMC11637401; doi:10.1371/journal.pone.0313661)

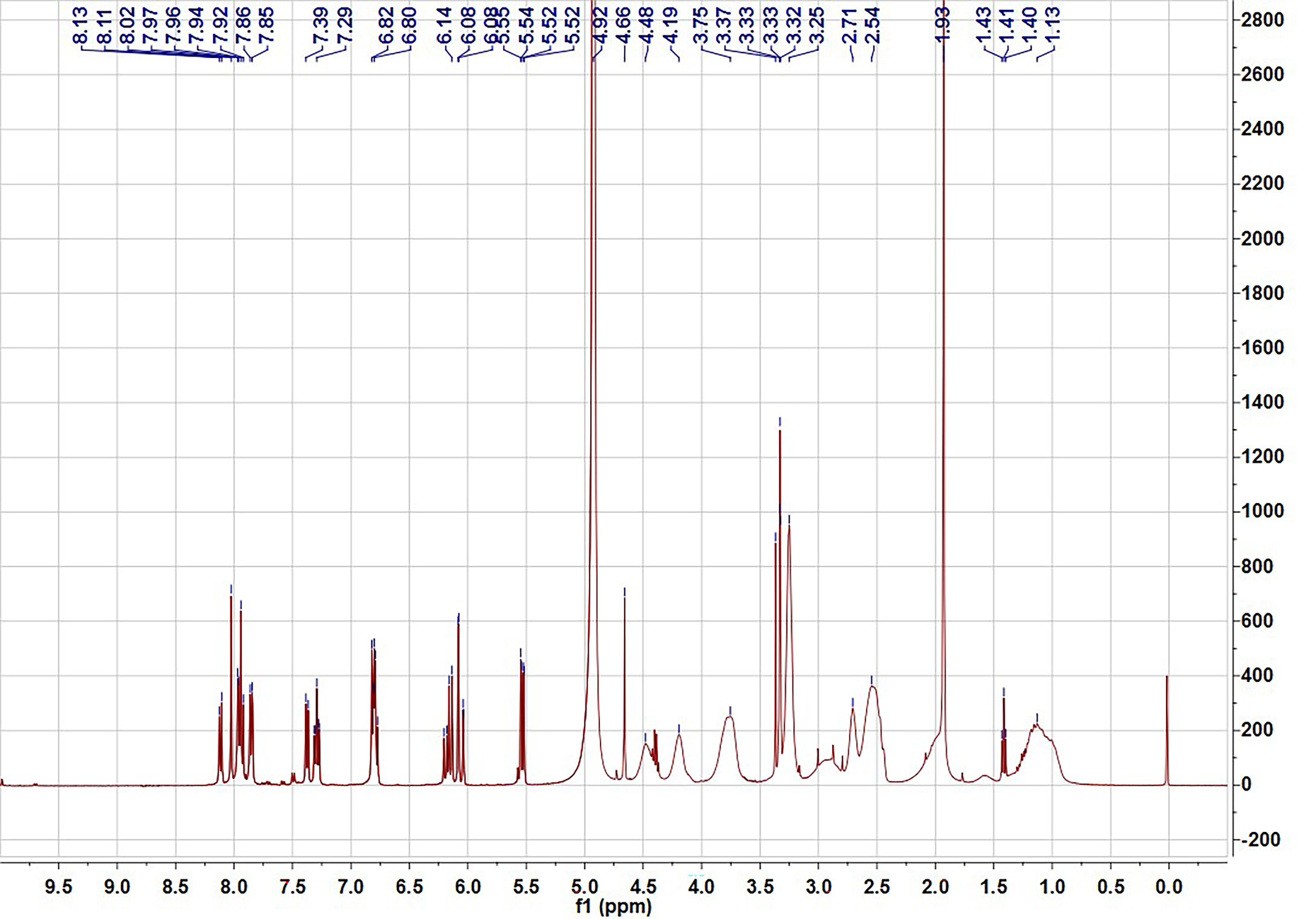

Supplement: S1 Fig — (TIF) [file pone.0313661.s001.tif]

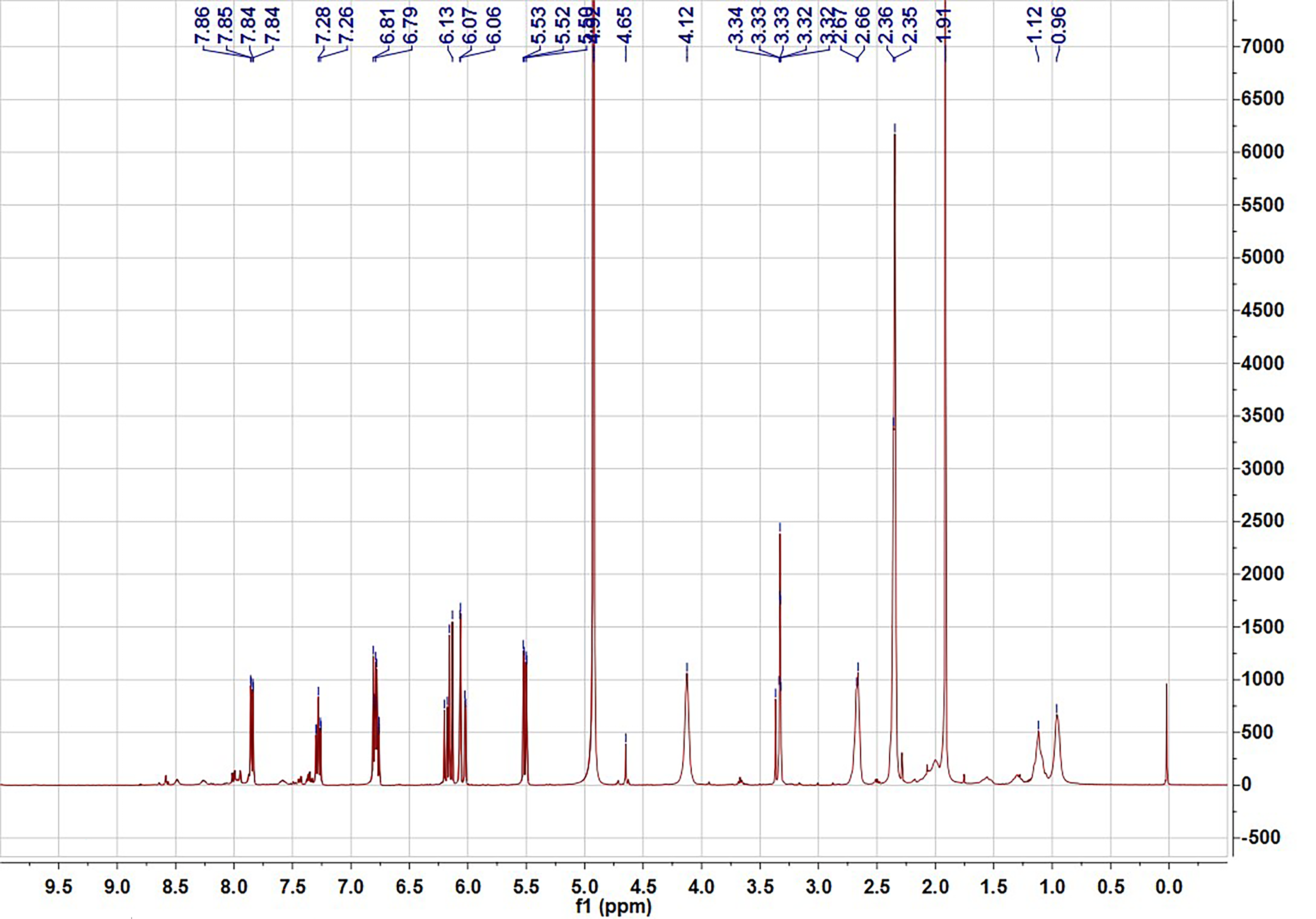

Supplement: S2 Fig — (TIF) [file pone.0313661.s002.tif]

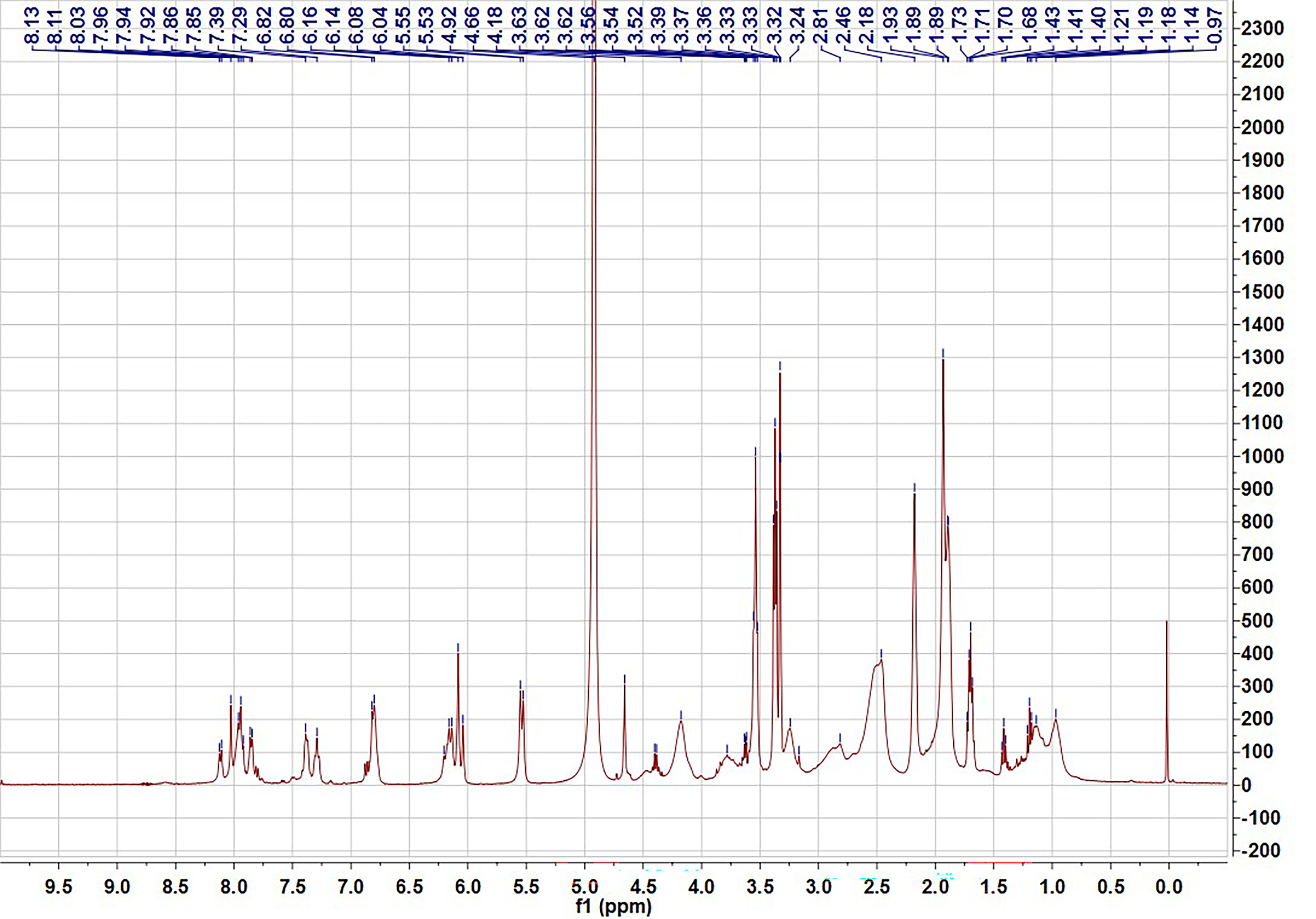

Supplement: S3 Fig — (TIF) [file pone.0313661.s003.tif]

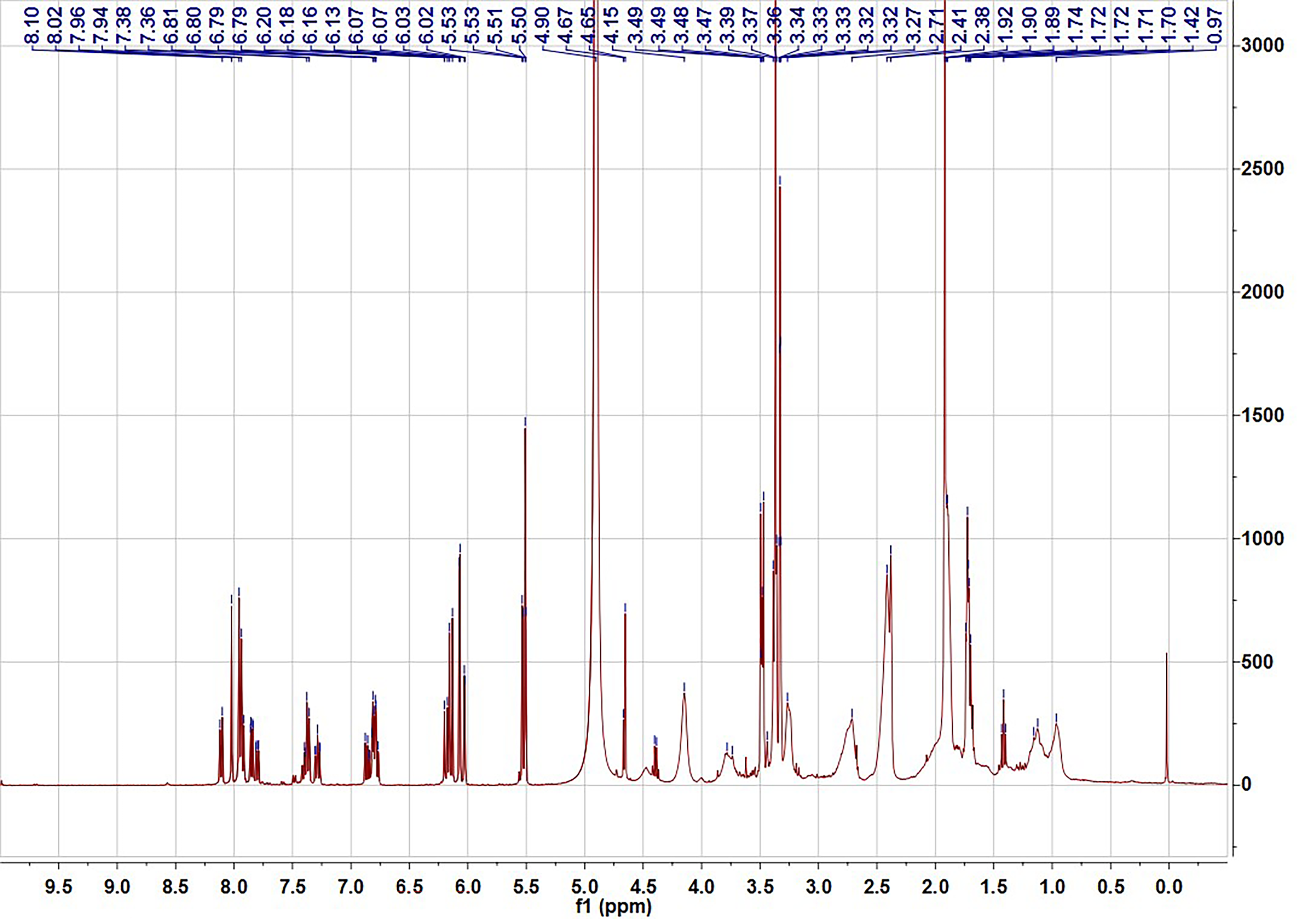

Supplement: S4 Fig — (TIF) [file pone.0313661.s004.tif]

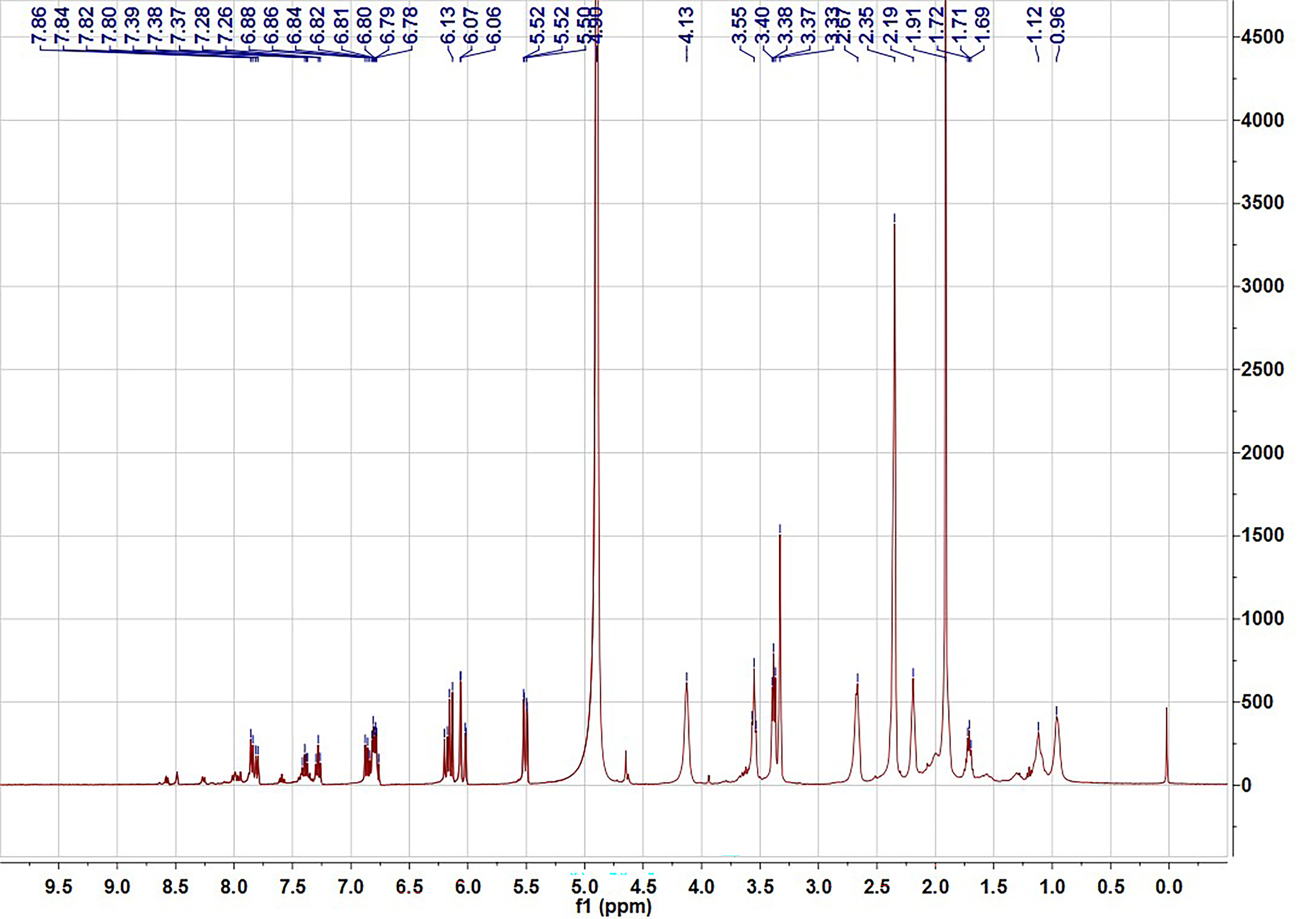

Supplement: S5 Fig — (TIF) [file pone.0313661.s005.tif]

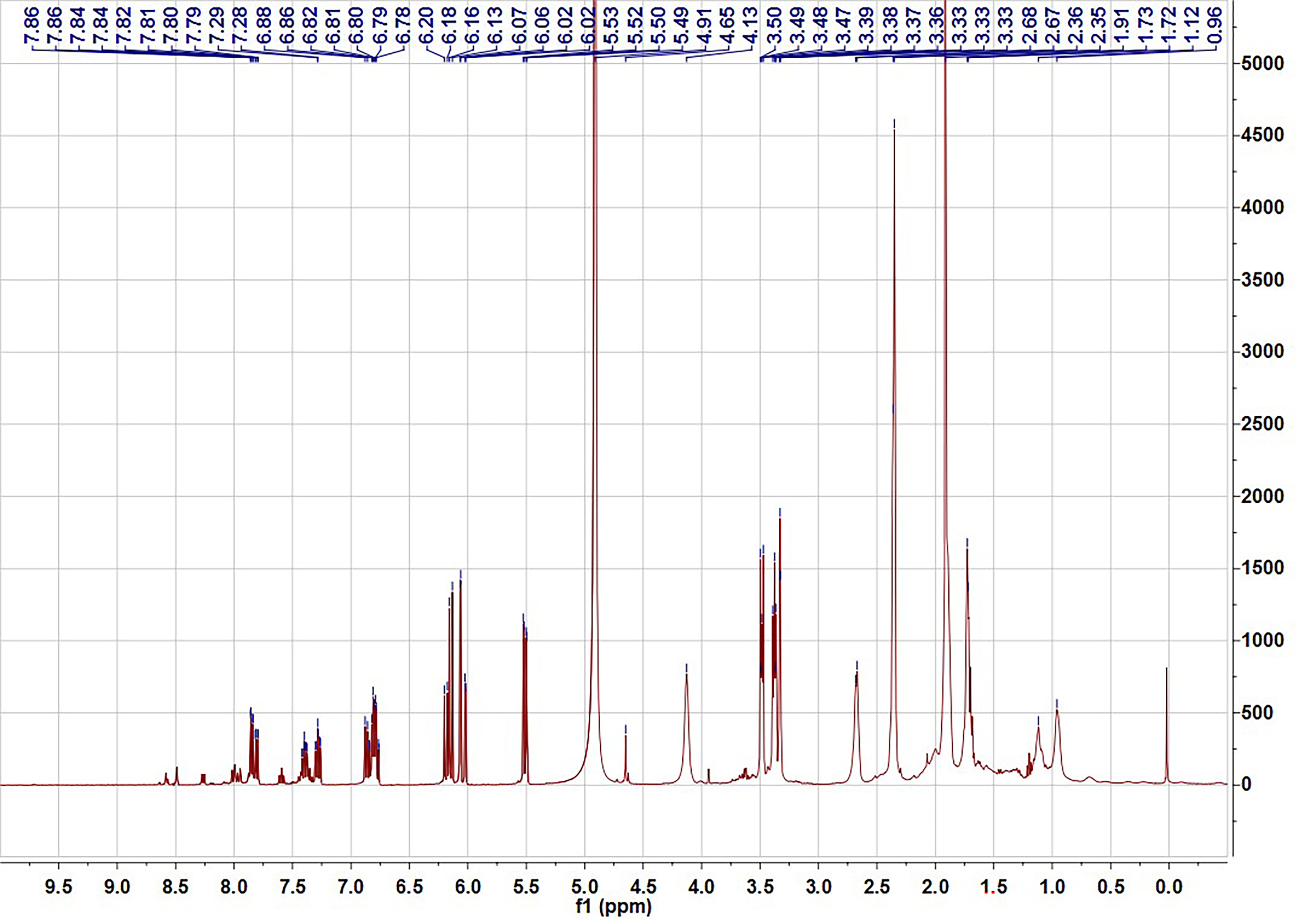

Supplement: S6 Fig — (TIF) [file pone.0313661.s006.tif]

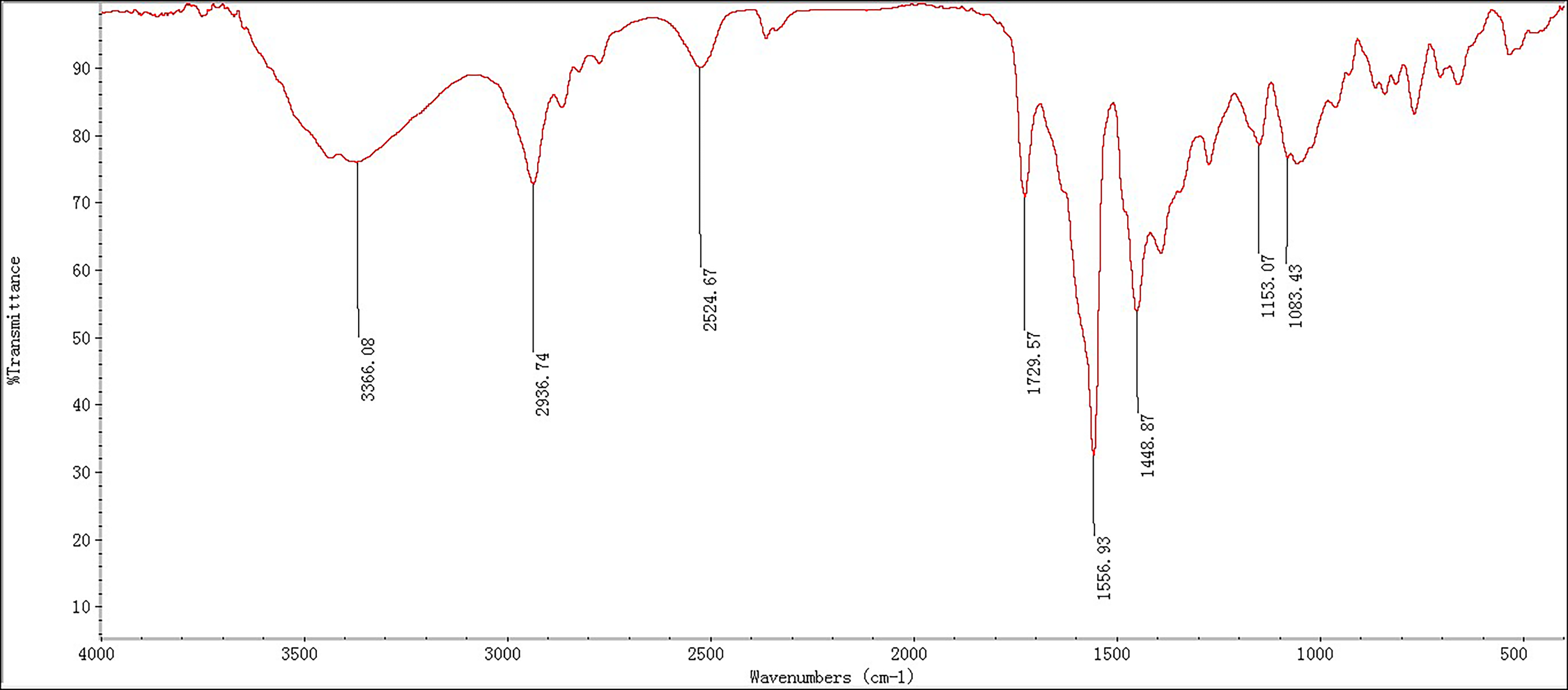

Supplement: S7 Fig — (TIF) [file pone.0313661.s007.tif]

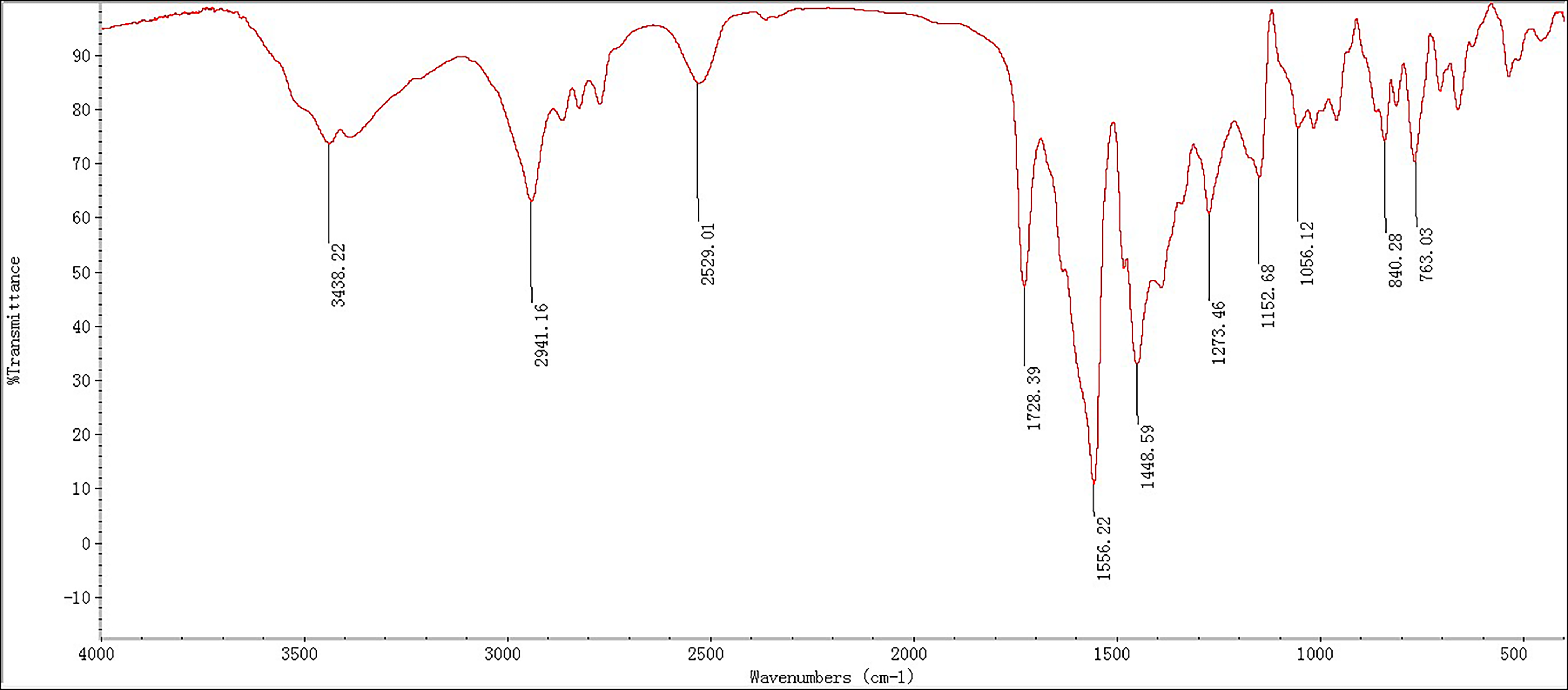

Supplement: S8 Fig — (TIF) [file pone.0313661.s008.tif]

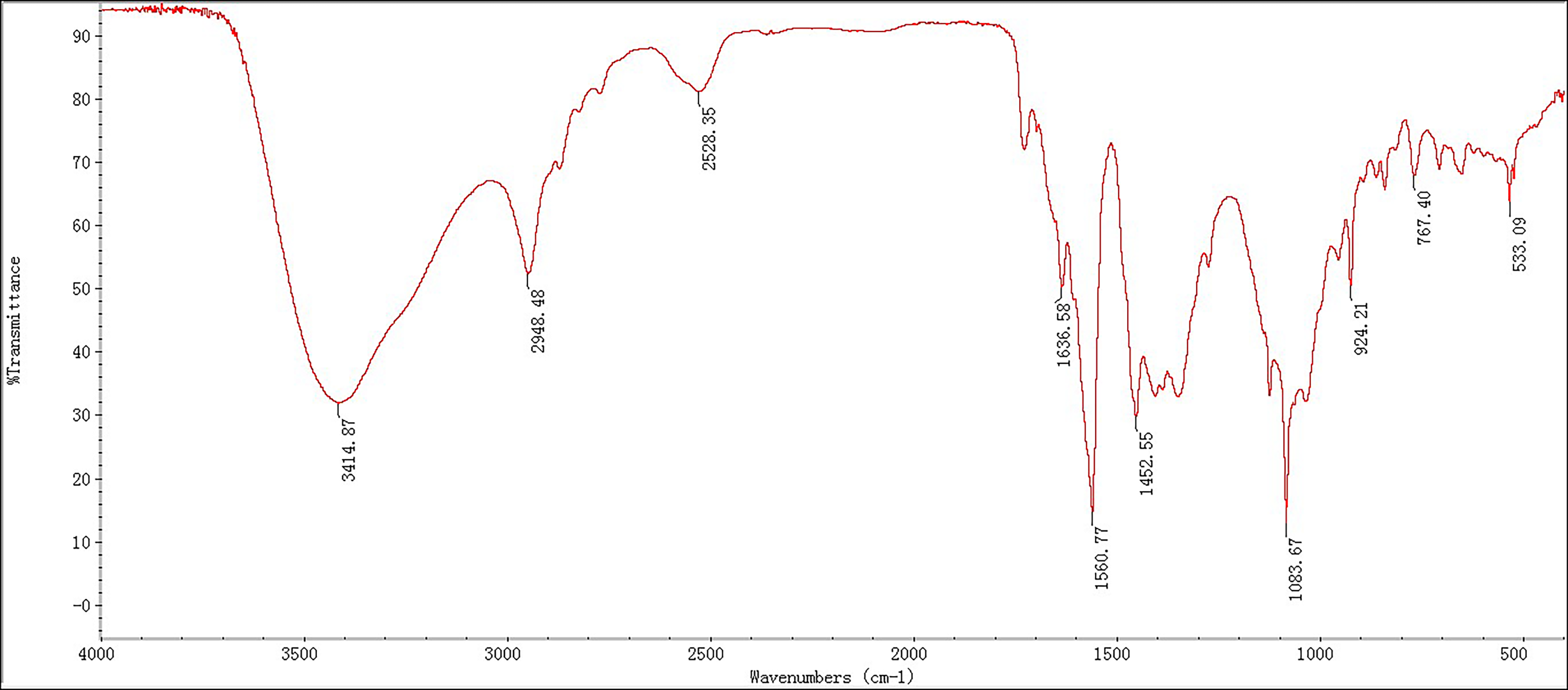

Supplement: S9 Fig — (TIF) [file pone.0313661.s009.tif]

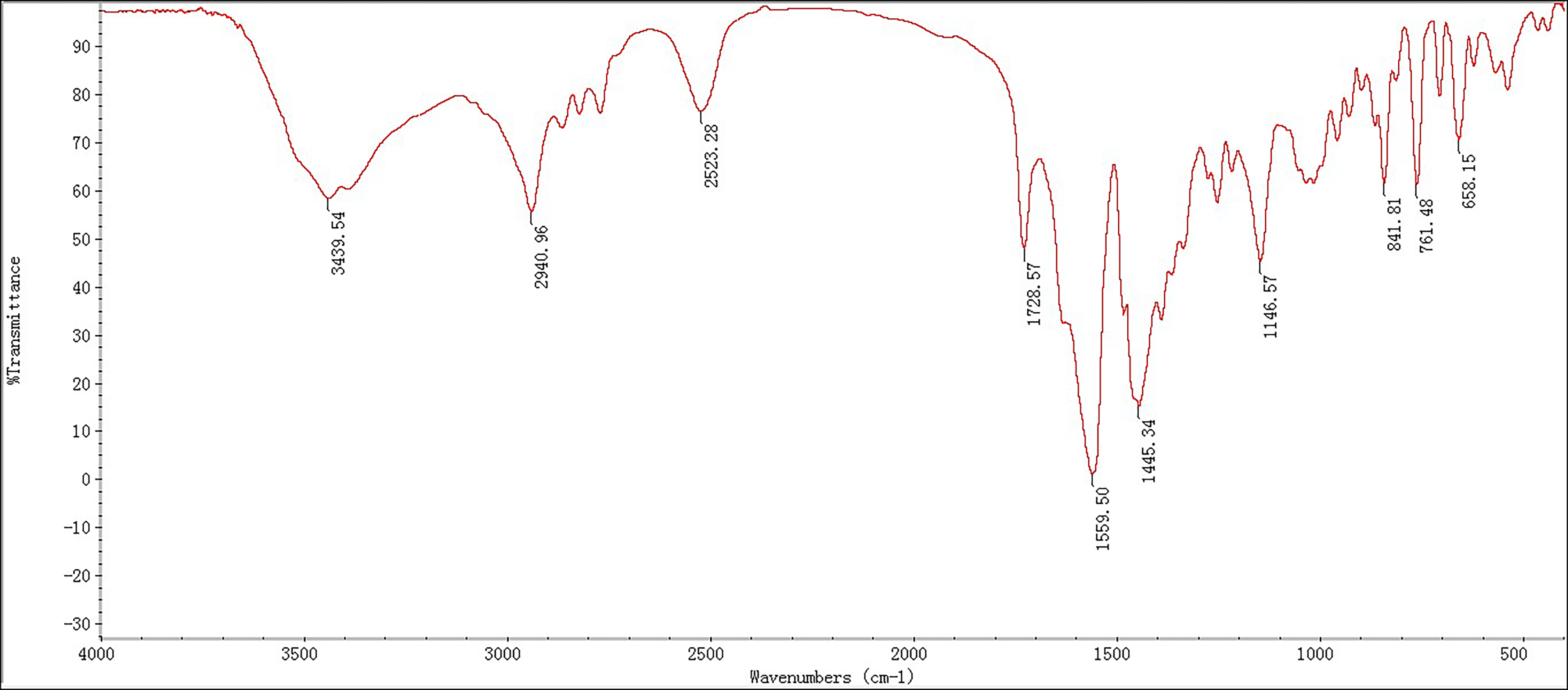

Supplement: S10 Fig — (TIF) [file pone.0313661.s010.tif]
